# Impact of Severity of Maternal COVID-19 Infection on Perinatal Outcome and Vertical Transmission Risk: An Ambispective Study From North India

**DOI:** 10.7759/cureus.21820

**Published:** 2022-02-01

**Authors:** Ritu Sharma, Ruchi Verma, Hariom K Solanki, Shikha Seth, Neha Mishra, Rakhee Sharma, Pinky Mishra, Monika Singh

**Affiliations:** 1 Obstetrics and Gynaecology, Government Institute of Medical Sciences, Greater Noida, IND; 2 Community Medicine, Government Institute of Medical Sciences, Greater Noida, IND; 3 Obstetrics and Gynaecology, Noida International Institute of Medical Sciences, Greater Noida, IND

**Keywords:** vertical transmission, perinatal outcome, maternal outcome, d-dimer, crp, severe maternal sars-cov-2 infection, covid wave

## Abstract

Background

In contrast to the first wave, the second COVID-19 wave has taken a huge toll affecting maternal outcomes adversely. The aim of this study was to investigate the consequences of the severity of maternal disease on perinatal outcomes and the risk of vertical transmission and to find out the factors associated with adverse fetomaternal outcomes.

Materials and methods

This was an ambispective observational study including COVID-19 infected pregnant patients; 20-40 years of age irrespective of gestational age admitted at Government Institute of Medical Sciences, UP, India. The patients were divided into two groups: CW 1 (COVID-19 Wave 1): Patients admitted between April 1, 2020 and December 31, 2020 and CW 2 (COVID-19 Wave 2): Patients admitted between April 1, 2021 to May 31, 2021.

Data in two groups were compared and analyzed with respect to the clinical profile, laboratory parameters, fetomaternal outcome and the risk of vertical transmission of COVID-19 infection.

Results

We included 134 eligible patients in the CW1 group and 58 in the CW2 group. Significantly more patients were symptomatic in CW2 (23.1% versus 60.3%, p= <0.001). In CW2, maternal neutrophil-lymphocyte ratio (NLR), C-reactive protein (CRP) and D-Dimer were significantly raised along with abnormal chest x-rays. There was a significant increase in maternal mortality in CW2 (1.5% vs 13.7%; p≤0.001).

A total of 76 patients delivered in CW1 and 26 in CW2 with increased incidence of cesarean section (43.4%; 42.3%), preterm deliveries (28.2%; 37%) and low birth weight (34.6%; 25.9%) in both waves, the difference among two groups being statistically insignificant. Compared to CW1, perinatal mortality was significantly increased in CW2 (2.2% vs 15.5%; p<0.001). Though nasopharyngeal swab tested positive in four neonates in CW1 and two neonates in CW2, no evidence of vertical transmission was observed even with increased severity of maternal illness. On regression analysis, D-Dimer and CRP were found to have a positive association with maternal and perinatal mortality.

Conclusion

The severity of maternal illness proportionately affects the neonatal outcome with no impact on the risk of vertical transmission of infection. D-Dimer and CRP have emerged as independent predictors for maternal and perinatal mortality and hence can be utilized in obstetrics decision-making.

## Introduction

COVID-19 infection has hit the whole world so hard that all have been left aghast. The pandemic has directly affected pregnant patients via COVID-19 infection as well as indirectly via disrupting maternal health services. The pathophysiology of the disease in pregnant women still needs to be explored as the fetomaternal outcome remains unpredictable. The dilemma has further been added by the ruthless second wave in India. During the first wave, most of the studies reported asymptomatic or mild infection in pregnant patients with low mortality; contrary to this, second wave has taken a huge death toll in India and other countries probably owing to the high transmission rate and rapid emergence of new viral strains [1]. In South Africa, 22.7% increase in maternal mortality, 4.8% increase in neonatal mortality and 3.4% increase in perinatal mortality have been reported [2]. India has also experienced 23% increase instead of 5.5% annual decline in maternal mortality ratio during a pandemic, which should draw urgent attention of policymakers [3,4]. The disproportionate impact of pandemic on maternal and perinatal outcome has surfaced in two waves worldwide as well as between high-income countries and low-middle income countries [5]. With this trend observed there has emerged need to recognize the impact of maternal disease severity on perinatal outcome with respect to morbidity, mortality and vertical transmission which will aid in better management of obstetrical patients. Currently a significant gap exists in understanding this correlation.

Predicting the third wave of the pandemic, decoding the factors related to maternal affliction is required to improve fetomaternal outcomes. Addressing this knowledge gap will help India and other countries to avoid striking down the achieved reduction in infant and maternal mortality rates. Due to limited data available from second wave and dearth of studies comparing the impact of maternal disease severity on perinatal outcome in two waves, we planned to investigate the added brunt on pregnant women infected with COVID-19 so as to ensure better maternal care in future waves, if any.

Objectives

The main objectives are to investigate the consequences of severity of maternal COVID-19 disease on perinatal outcome and on the risk of vertical transmission, and to find out the factors associated with adverse fetomaternal outcomes.

## Materials and methods

This was a single-center ambispective observational cohort study carried out at the tertiary-care institute, Government Institute of Medical Sciences, UP, India, after obtaining the ethical clearance (GIMS/IEC/HR/2021/30). We included all eligible pregnant patients with COVID-19 infection hospitalized from April 2020 to May 2021.

All pregnant patients with RTPCR confirmed SARS-CoV-2 infection, 20-40 years of age and consent to participate were included in the study irrespective of gestational age. Pregnant patients without RTPCR confirmed SARS-CoV-2 infection, postpartum patients and aged <20/>40 years were excluded from the study. Depending on the wave, the participants were divided into two groups: CW 1 (COVID-19 Wave 1): Patients admitted between April 1, 2020 and December 31, 2020 and CW 2 (COVID-19 Wave 2): Patients admitted between April 1, 2021 and May 31, 2021.

A detailed history was taken and an examination was done. Blood samples were collected for baseline hematological investigations (CBC, LFT, KFT); inflammatory (C-reactive protein [CRP], D-dimer, serum ferritin) and coagulation markers (D-dimer, serum fibrinogen, PT/INR, LDH). Chest X-ray with the abdominal shield was done where indicated. All patients were managed as per the standard obstetrical guidelines [6]. For evaluating the risk of vertical transmission of the infection, various biological samples for COVID-19 RT PCR test (vaginal swab, amniotic fluid, placental swab, cord blood, peritoneal fluid and breast milk) and cord blood sample for additional IgM test were collected from the cases who delivered while being admitted in the hospital during active infection. The nasopharyngeal swab of the neonate was collected at 24 hours of birth. The perinatal-maternal outcome in two groups was recorded. Data in two groups were compared and analyzed with respect to the clinical profile, laboratory parameters, fetomaternal outcome and the risk of vertical transmission of COVID-19 infection.

**Figure 1:**
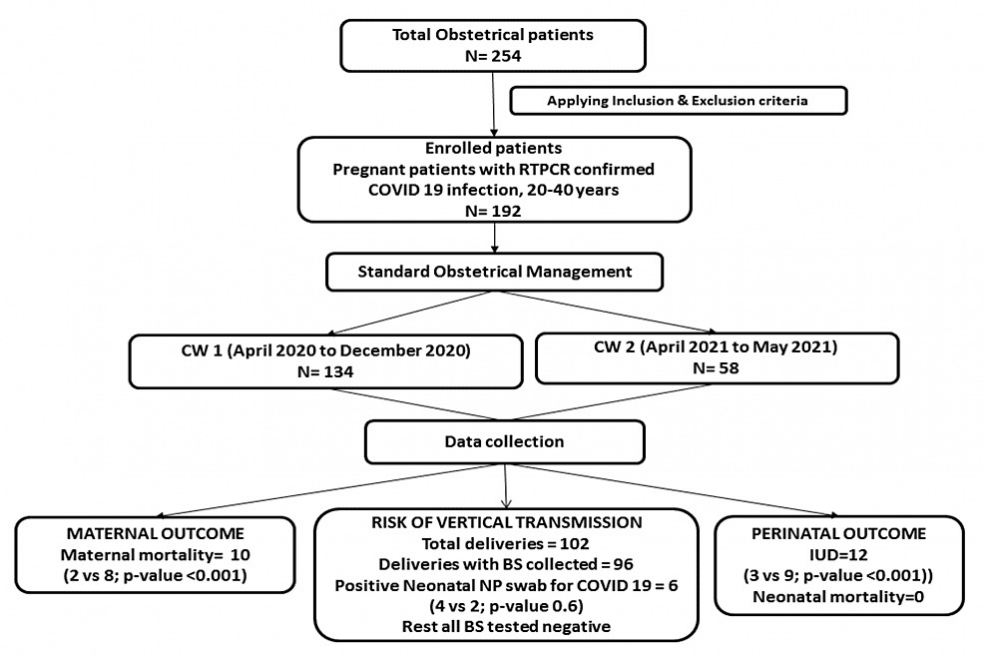
Study design and primary study outcomes BS, Biological samples; NP, nasopharyngeal; IUD, intrauterine death

The primary outcome measures were maternal mortality, perinatal mortality and evidence of vertical transmission (Figure 1). Secondary outcome measures included maternal clinical and laboratory profile, therapeutic interventions, obstetrical outcome and neonatal profile.

Statistical analysis

Continuous variables were presented as mean ± standard deviation (SD) or median with range, while categorical variables were presented as numbers and percentages (%). The student’s t-test was used to compare the normally distributed data. We also used the Chi-square test and Mann-Whitney U test as per requirement. P-value < 0.05 was considered statistically significant. Regression models were used to assess risk factors. We used SPSS 21.00 software (IBM Corp, Armonk, NY, USA) for statistical analysis.

## Results

During the study period, 254 obstetrical patients with COVID-19 infection were admitted to the hospital; out of the 192 were enrolled in the study considering inclusion and exclusion criteria with 134 patients in the CW-1 group while the CW-2 group included 58 patients (Figure 1).

Obstetrical profile in two waves

In the first wave (of nine months duration) average admission rate was 14.8/month while in the second wave (of two months duration) it was 29/month. Though the first wave mainly affected younger obstetrical patients (<30 years), the mean age in the two waves was statistically comparable (p=0.13). The two groups were comparable with respect to socioeconomic status, gestational age, gravidity and the presence of co-morbidities. Compared to CW1, significantly more pregnant patients were symptomatic in CW2 (23.1% versus 60.3%, p≤0.001) with moderate to severe disease at admission; also cough (p<0.001), shortness of breath (p<0.001) and fever (p=0.011) were significantly more common in CW2 (Table 1).

**Table 1:** Obstetrical profile in two waves SOB: Shortness of breath; NLR: Neutrophil-lymphocyte ratio; PLR: Platelet-lymphocyte ratio; CRP: C-reactive protein; INR: International normalized ratio

Variable		CW 1 (N=134) n (%)	CW 2 (N=58) n (%)	P-value
Age (years)	(Mean ± SD)	26.8±4.8	28.1±5.4	0.13
	<30 yrs	106 (79.1)	37 (63.7)	0.03
	>30yrs	28 (20.8)	21 (36.2)	
Socioeconomic status	Lower	47 (35.0)	23 (39.6)	0.79
	Middle	71 (52.9)	27 (46.5)	
	Upper	16 (11.9)	8 (13.7)	
Gestational age (weeks)	(Mean ± SD)	33.3±7.8	31.7±8.3	0.12
	<12 weeks	3 (2.2)	3 (5.1)	0.13
	12-28 weeks	22(16.4)	13(22.4)	
	>28 weeks	109 (81.3)	42 (72.4)	
Gravidity	G1	55 (41)	27 (46.5)	0.5
	G2	39 (29.1)	16 (27.5)	
	>G3	40 (29.8)	15 (25.8)	
Co morbidities	Present	22 (16.4)	12 (20.7)	0.6
Clinical presentation	Symptomatic	31 (23.1)	35 (60.3)	<0.001
Severity	Mild	26 (19.4)	15 (25.8)	0.09
	Moderate	3 (2.2)	5 (8.62)	0.023
	Severe	2 (1.5)	15 (25.8)	<0.001
Symptoms	Cough	10 (7.4)	22 (37.9)	<0.001
	Fever	21 (15.6)	19 (32.7)	0.011
	SOB	2 (1.5)	19 (32.7)	<0.001
Laboratory Profile				
Investigations		CW1 Median (Range)	Median (Range)	P-value
Haemoglobin (gm/dL)		10.9 (6.8-14.4)	11.05 (8.6-14.9)	0.2
TLC (cells/mm^3^)		8,800 (3,800-32,500)	9,500 (1,100-44,310)	0.35
NLR		3.9 (1.25-18.2)	5.6 (1.1-33.5)	0.002
PLR		0.09 (0.02-0.77)	0.11 (0.02-0.4)	0.15
S. Ferritin (ng/ml)		50.7 (3-387)	133.8 (1-1161)	<0.001
CRP (mg/l)		6.7 (0.03-59.5)	20.2 (1.1-137.5)	<0.001
INR		1.02 (0.75-1.92)	1.1 (0.75-4.8)	<0.001
D-Dimer (µg/ml)		1.38 (0.11-10)	2.1 (0.15-9.95)	0.002
S. Fibrinogen (mg/dL)		433.6 (11.8-996)	365.0 (301-601)	<0.001
LDH (U/L)		337.4 (0.3-1,764)	440.5 (255-5,394)	<0.001
X ray		N=131	N=55	
Abnormal X-ray findings		5 (3.8)	24 (41.3)	<0.001

Compared to CW1, in CW2 neutrophil-lymphocyte ratio (NLR), CRP, serum ferritin, D-dimer, serum fibrinogen and LDH were significantly raised. The median values of CRP and D-dimer in CW1 and CW2 were 6.7 vs 20.2mg/L and 1.38 vs 2.1 µg/mL, respectively. More patients in CW2 had abnormal x-ray findings as well (3.8% vs 41.3%; p<0.001) (Table 1).

Maternal and perinatal outcome in two waves

It was observed that more patients in CW2 required oxygen support, ICU admission and ventilation than patients in CW1, the difference being statistically significant (p<0.001). Also, in CW2, the requirement of steroids and low molecular weight heparin (LMWH) was significantly increased (4.4% vs 51.7%, p<0.001; 11.1% vs 41.3%, p<0.001, respectively). Nine patients received remdesivir and one patient even received tocilizumab in CW2. Duration of mean hospital stay was comparable in two waves (p=0.77). There were two maternal deaths in CW1 as compared to eight in CW2 with significantly increased maternal mortality in CW2 (p<0.001). Overall, six patients died antenatally in two waves. The most common causes of maternal mortality included acute respiratory distress syndrome (ARDS) and disseminated intravascular coagulation (DIC).

There were 76 deliveries (with two twin deliveries) in CW1 and 26 in CW2 (with one twin delivery). Cesarean section rate (43.4%; 42.3%) and preterm delivery rates (28.2%; 37%) were higher in two waves than that in the general population. There was no difference among the two waves with respect to low birth weight (LBW) and neonatal intensive care unit (NICU) admission; however, the incidence of LBW was increased in both waves (34.6%; 25.9%). Compared to CW1, perinatal mortality was significantly increased in CW2 (2.2% vs 15.5%; p<0.001). There was no neonatal mortality observed in either wave (Table 2).

**Table 2:** Maternal and perinatal outcome in two waves *One twin delivery; **two twin deliveries; LMWH, low molecular weight Heparin; LBW, low birth weight

Variable	CW 1 n (%)	CW 2 n (%)	P-value
MATERNAL OUTCOME			
	N=134	N=58	
ICU admission	5(3.7)	23(39.6)	<0.001
Oxygen support	5(3.7)	20 (33)	<0.001
Ventilator	2(1.5)	9(15.5)	<0.001
Steroid	6(4.4)	30(51.7)	<0.001
LMWH	15(11.1)	24(41.3)	<0.001
Hydroxychloroquine	131 (97.7)	55 (94.8)	0.369
Remdesivir	0(0)	9(15.5)	<0.001
Tocilizumab	0	1(1.7)	0.3
Hospital stay (days) Mean ± SD	7.6±4.3	7.6±5.2	0.77
Maternal mortality	2(1.5) Antenatal 1; Postnatal 1	8(13.7) Antenatal 5; Postnatal 3	<0.001
Total deliveries	N=76**	N= 26*	0.16
Cesarean delivery	33(43.4)	11(42.3)	0.06
NEONATAL OUTCOME			
Variable	N=78	N=27	
Preterm<37 weeks	22(28.2)	10(37.0)	0.46
Live- delivered	76 (56.7)	23 (39.6)	< 0.001
Perinatal Mortality	3 (2.2) Delivered 2; Undelivered 1	9 (15.5) Delivered 4; Undelivered 5	
Male	49 (62.8)	20 (74)	0.5
Apgar Score 5min <7	6 (7.7)	2 (7.4)	>0.9
LBW <2.5Kg	27 (34.6)	7 (25.9)	0.48
NICU admission	13 (18.5)	8 (33.3)	0.08
Room in & Breastfed	36 (47.3)	9 (33.3)	0.09
Neonatal Mortality	0 (0)	0 (0)	0 (0)
Neonatal COVID-19	4 (5.2)	2 (8.6)	0.6

Vertical transmission

**Figure 2:**
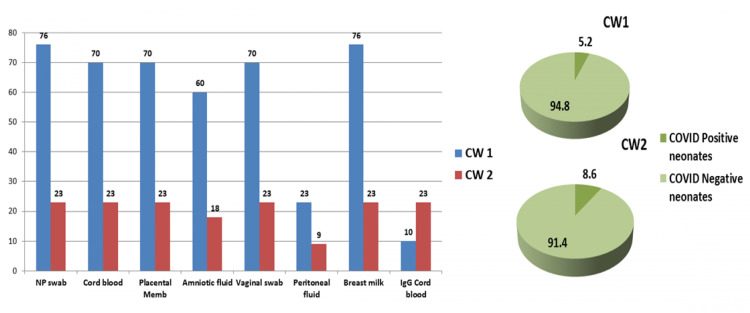
Bar diagram shows the number of RT-PCR tests and IgM tests of various biological samples to assess the risk of vertical transmission (except nasopharyngeal swabs, all samples tested negative). Pie chart shows the results of neonatal RT-PCR from nasopharyngeal (NP) swabs.

For assessing the risk of vertical transmission, apart from nasopharyngeal swabs of neonates, we also collected vaginal swabs, amniotic fluid, placental swabs, cord blood, peritoneal fluid and breast milk for RT-PCR test of COVID-19. The cord blood sample was also subjected to IgM testing. Out of 99 neonates, nasopharyngeal swab tested positive in four (5.2%) neonates in CW1 & two (8.6%) neonates in CW2 (p=0.6); the rest of all biological samples tested negative in both waves (Figure 2). In CW1, 47% of neonates and in CW2, 33.3% of neonates were roomed-in and breast-fed. All positive neonates remained asymptomatic till the hospital stay.

Regression analysis

To get adjusted estimates for maternal and perinatal mortality, we applied binary logistic regression, backward LR including variables - age, period of gestation (POG), co-morbidities, NLR, CRP, D-dimer, LDH, chest x-ray, gravidity, mode of delivery and NICU admission. The probabilities for entering a variable in the equation were kept at 0.05 and to exclude from the equation was kept at 0.25. CRP and D-Dimer were found to be significantly associated with maternal mortality on a combined analysis of two waves (OR 1.079, 95% CI 1.017-1.146; p 0.012 and OR 2.56, 95% CI 1.071-6.119; p 0.035, respectively) (Table 3).

**Table 3:** Binary logistic regression (backward) to assess risk factors for maternal mortality POG: period of gestation; CRP: C-reactive protein

	B	S.E.	Exp (B)	95% CI	P-value
Age	1.991	1.374	7.322	0.496-108.163	0.147
POG (weeks)	-0.319	0.223	0.727	0.47-1.125	0.152
Mode of delivery	1.515	1.368	4.548	0.311-66.437	0.268
CRP	0.076	0.030	1.079	1.017- 1.146	0.012
D-Dimer	0.94	0.445	2.56	1.071- 6.119	0.035
Constant	-5.504	3.581	0.004		0.124

D-dimer again showed a significant association with perinatal mortality on combined analysis (CW1 and CW2) (OR 1.446, 95% CI 1.04-2.01; p 0.028). A positive association of perinatal mortality was observed with maternal mortality as well (Table 4).

**Table 4:** Binary logistic regression (backward) to assess risk factors for perinatal mortality PLR: Platelet-lymphocyte ratio; CRP: C-reactive protein

	B	S.E.	Exp (B)	95% CI	P-value
Maternal mortality	5.173	1.349	176.461	12.5332-2482.562	<0.001
PLR	-6.89	4.002	0.001	0-2.595	0.085
D-Dimer	0.386	0.168	1.446	1.04-2.01	0.028
Mode of Delivery	-0.811	0.646	0.444	0.125-1.575	0.209
Constant	-3.481	0.778	0.031		<0.001

Similarly, we tried to find out the association of severity of maternal illness, mode of delivery and rooming-in with the risk of vertical transmission but could not find any association among them.

## Discussion

It is evident that the COVID-19 pandemic has adversely affected reproductive health services throughout the world with variation in the impact of the two waves. We have tried to explore the impact of severity of maternal COVID-19 infection on perinatal outcome and the risk of perinatal transmission which is still a grey zone.

In our study the mean gestational age in two waves was comparable (33.3±7.8 and 31.7±8.3 weeks; p=0.12). Mahajan et al. also did not observe any significant difference in the gestational age among two waves (36 weeks vs 32; p=0.157) [7]. Similar to our study, other studies also registered more symptomatic patients in wave 2 (14.2% vs 28.7%) [8] and reported fever, cough and shortness of breath as the most common symptoms in two waves [9,10]. 

While evaluating the laboratory profile we observed significantly raised NLR (p=0.002), CRP (p<0.001), D-Dimer (p=0.002) and abnormal chest X-ray findings (p<0.001) in CW2 obstetrical patients. Lombardi et al. also noted raised median values of NLR, CRP and D-dimer (4.4, 1.66 mg/dL and 1,727 μg/L, respectively) [11]. The umbrella review by A Ciapponii including 66 systematic reviews of observational studies related to COVID-19 in pregnancy reported raised CRP in 28%-96%, lymphocytopenia 33.6% to 80% and abnormal radiological findings in 7.1% to 99% cases [12]. Lymphocytopenia and CRP were reported as most common lab findings by others as well [9]. 

Depending on the evidence available there was a shift from hydroxychloroquine in the first wave to remdesivir, tocilizumab and steroids in the second wave [10,13,14]. In our study, we supplemented hydroxychloroquine with other drugs in the treatment protocol as per the recommendations from the national organization [8]. A single centre observational study from Belgium reported significantly increased use of dexamethasone (3.2% vs 61%, p<0.001); high flow nasal oxygen (2% vs 12%, p<0.0001) and remdesivir (0.6% vs 13.3%, p 0.0001) in second wave [15]. We also observed the same. As in other studies, we also registered a significant increase in the use of non-invasive ventilation, LMWH and steroids in the second wave [10,14]. These therapeutic interventions resulted in decreased mortality in some studies which was not observed in our study [15].

We observed significantly increased severe infection (1.5% vs 25.8%, p<0.001), ICU admission (3.7% vs 39.6%, p<0.001) and increased maternal mortality (1.5% vs 13.7%, p<0.001) in CW2. Mahajan et al. from Mumbai, India also reported significantly more severe infection (1.7% vs 8.5%, p<0.001); more ICU admissions (2.4% vs 11.6%, p<0.001); increased case fatality rate(CFR) (0.7% vs 5.7%, p<0.001) and eightfold increase in maternal mortality ratio (MMR) (10.2/1,000 births vs 83.3/1,000 births, p<0.001) in wave 2 [8]. The odds ratio (OR) of receiving invasive ventilation for COVID-19 versus non-COVID-19 pregnant women has been reported as 1.88 by Ciapponii et al. (95% confidence interval [CI] 1.36-2.60) [12]. Kadiwar et al. reported that the ECMO referrals for peripartum patients were significantly more common in the second wave (12% vs 31%; p=0·047) [1]. In our study, we observed a statistically significant increase in maternal mortality (1.5% vs 13.7%; p<0.001) and noted ARDS and DIC as the most common cause of death. Mahajan et al. reported pneumonia and respiratory failure as the most common causes of maternal mortality [8]; while Nair et al. attributed the increase in overall case fatality rate to reduced hospital visits and reduced hospital births rather than the COVID-19 infection directly [3]. Contrary to our findings studies from Spain reported better outcomes in the second wave with a lower case fatality rate (24.0% vs 13.2%) which was attributed to more experience, availability of more effective therapeutic interventions and probably to a new variant of concern (VOC) 20A.EU1 [10,16]. In India new VOC, B.1.617.2/Delta variant is reported to be responsible for the second wave that resulted in increased severity and poor outcome [17,18].

We observed comparable rates of cesarean delivery and preterm delivery in two waves which however were higher than that in the general population. Mahajan et al. too observed that the two waves were comparable with respect to cesarean deliveries (39.6% vs 37.1%), preterm birth rate/1,000births (93.2 vs 128.7, p 0.09) and stillbirth rate/1,000births (15.3 vs 34.1, p 0.06) [8]. Another study from India reported an overall preterm delivery rate of 11.3% with an increase in the second wave (10.2% vs 14%, p=0.065) [19]. Gurol-Urganci et al. conducted a population-based cohort study in England and reported a twofold increase in preterm delivery (12.1% vs 5.8%; aOR 2.17; 95% CI, 1.96-2.42; p<0.001) among COVID-19 infected mothers. They also observed that among the neonates born to COVID-19 infected mothers the adverse outcome (5.2% vs 7.6%; aOR 1.45; 95% CI, 1.27-1.66; p<0.001), NICU admission (10.7% vs 13.7%; aOR 1.24; 95% CI, 1.02-1.51; p=0.03), and prolonged hospital stay (18.0% vs 27.6%; aOR 1.61; 95% CI, 1.49-1.75; p<0.001) were significantly higher as compared to those born to non-infected mothers. However, once analysis was limited to term pregnancies only, no significant difference was observed among the two groups. The authors inferred that preterm birth secondary to maternal COVID-19 infection was primarily responsible for adverse neonatal outcomes [20]. Similar was the inference from other studies [21,22]. The observed odds ratio of NICU admission among neonates born to COVID-19 infected versus non-infected mothers was 3.13 (95%CI 2.05-4.78) [12]. We reported an increased incidence of NICU admission in wave 2 (18.5% vs 33.3%; p=0.08) and most of these admissions were for the purpose of isolation only.

With the increased severity of maternal disease, we found a significant rise in perinatal mortality in CW2 (2.2% vs 15.5%, p=0.001). A study from India too reported significant increase in stillbirth rate (1.5% vs 3.6%, p=0.025) with associated increase in severe infection (0% vs 33.3%) [7]. The rate of intrauterine demise among the women with active infection was reported to be almost double as compared to those without infection (8.5/1,000 versus 3.4/1,000; aOR 2.21; 95% CI, 1.58-3.11; p<0.001) [20]. The reported incidence of neonatal mortality ranges from 0% to 9.2% [12]; we did not observe any neonatal death. Chmielewska et al. after conducting a systematic review and meta-analysis observed that maternal mortality and stillbirth were significantly increased during pandemics, especially in low-middle income countries. No change was observed in the overall incidence of preterm birth, NICU admission and neonatal death [5]. An observational analytical study on 1295 COVID-19 infected pregnant women, comparing two waves in Spain reported a strong correlation of maternal morbidity with perinatal morbidity similar to our study. On univariate analysis mechanical ventilation and maternal ICU admission showed a positive association with perinatal morbidity (10-fold and fivefold increased risk respectively); while lymphocytopenia was associated with maternal morbidity [23]. A study from India also attributed the increased perinatal mortality directly to increased severity of maternal infection, and indirectly to increased obstetrical complications due to disrupted maternal services [7].

There are limited studies evaluating the role of inflammatory markers in predicting the prognosis of COVID-19 infection among obstetrical patients. Lombardi et al. correlated the admission values of various biochemical and inflammatory markers in COVID-19-infected obstetrical patients with the requirement for oxygen supplementation. They observed that for every 1,000 lymphocyte cells decrease, the risk for oxygen supplementation increases by 26%. Since there is a physiological rise in D-dimer and a fall in serum Ferritin levels during pregnancy, they questioned their prognostic role and inferred that compared to D-dimer and ferritin, CRP is a reliable predictor for obstetrical outcome [11]. Bernard et al. again observed a positive association of CRP with mortality (HR 1.002, 95% CI 1.001-0.004, p 0.0064) [15]. In our study D-dimer has emerged as an independent predictor for perinatal as well as maternal mortality and CRP as an additional prognostic marker for maternal mortality; thereby establishing that both the markers are valuable predictors of perinatal and maternal outcomes. Therefore, our study concludes that the patient should be kept under close surveillance if either of the markers is raised.

The reported neonatal COVID-19 infection rate in the literature varies from <1% to 9.1% [5,24,25] and in our study, it was 5.7%. There is evidence of the expression of ACE2 receptors on the placenta, uterus and umbilical cord [26,27], so there was a need to investigate the possibility of vertical transmission via testing various biological samples apart from neonatal nasopharyngeal swabs. In our previous study including patients from the first wave, we could not find any evidence of vertical transmission after testing various biological samples [28]. Owing to the assumption that the severity of the maternal disease may increase the risk of vertical transmission, we planned to carry forward with the present study adding IgM testing on cord blood samples. In the present study also, we could not find any evidence of vertical transmission even with increased severity of maternal infection. We could not find any published study evaluating the correlation of severity with the risk of vertical transmission. Further, since all positive neonates in our study remained asymptomatic with repeat nasopharyngeal swabs 48 hours apart tested negative in the majority suggesting that positivity may be due to transient colonization and not a true infection.

In an observational prospective cohort study from New York by Salvatore including 120 neonates born to COVID-19 infected mothers, neither anyone tested positive at birth, nor even after being roomed-in and breast-fed among those who opted for it. The study concluded that the risk of perinatal transmission is negligible provided infection control practices are undertaken [29]. Our study too supported the same, as all neonates who were roomed-in and breastfed remained asymptomatic. In a multicenter cohort study in Massachusetts including 255 neonates born to infected mothers the incidence of neonatal COVID-19 infection was found to be 2.2% and the risk was directly associated with the maternal social vulnerability index (adjusted OR, 4.95; 95%CI, 1.53-16.01; p=0.008), with no association being observed with the mode of delivery, the severity of maternal disease and rooming-in [21]. In our study, we also could not find any such association.

Strengths and limitations

This is the first study from North India comparing maternal and perinatal profiles in two waves reflecting the impact of severity of COVID-19 infection. We tried to find the association of maternal and perinatal outcomes with other variables and used regression to adjust for heterogeneity. We included an adequate cohort of neonates with all possible biological samples tested for any evidence of vertical transmission. However, it was a single-center study, so the results may not be generalized. Also, we could not go for gene sequencing at our center to be sure of the VOC responsible for the second wave.

## Conclusions

The most virulent variant of concern of COVID-19 virus (VOC), B.1.617.2/Delta variant, has been held responsible for the ruthless second COVID-19 wave resulting in increased maternal and perinatal mortality. Our study concludes that the severity of maternal illness proportionately affects the neonatal outcome with no impact on the risk of vertical transmission of infection. D-dimer and CRP have emerged as independent predictors for maternal and perinatal mortality in our study. Since the emergence of the new VOC-Omicron the panic has again resurfaced, we want to disseminate the results of our study so that one can utilize simple, universally available investigations D-dimer and CRP along with maternal profile to review the treatment time and optimize the maternal and perinatal outcome.
